# Effect of different ankle joint positions on medial gastrocnemius muscle fiber strains during isometric plantarflexion

**DOI:** 10.1038/s41598-023-41127-z

**Published:** 2023-09-11

**Authors:** Brandon T. Cunnane, Usha Sinha, Vadim Malis, Ryan D. Hernandez, Edward Smitaman, Shantanu Sinha

**Affiliations:** 1 Physics, San Diego State University, San Diego, CA USA; 2 Muscle Imaging and Modeling Lab, Dept. of Radiology, UC San Diego, 8939 Villa La Jolla, San Diego, CA 92121 USA; 3 Dept. of Radiology, UC San Diego, San Diego, CA USA

**Keywords:** Health care, Medical imaging, Magnetic resonance imaging

## Abstract

Muscle force production is influenced by muscle fiber and aponeurosis architecture. This prospective cohort study utilizes special MR imaging sequences to examine the structure–function in-vivo in the Medial Gastrocnemius (MG) at three-ankle angles (dorsiflexion, plantar flexion—low and high) and two sub-maximal levels of maximum voluntary contraction (25% and 50%MVC). The study was performed on 6 young male participants. Muscle fiber and aponeurosis strain, fiber strain normalized to force, fiber length and pennation angle (at rest and peak contraction) were analyzed for statistical differences between ankle positions and %MVC. A two-way repeated measures ANOVA and post hoc Bonferroni-adjusted tests were conducted for normal data. A related samples test with Friedman’s 2-way ANOVA by ranks with corrections for multiple comparisons was conducted for non-normal data. The dorsiflexed ankle position generated significantly higher force with lower fiber strain than the plantarflexed positions. Sarcomere length extracted from muscle fiber length at each ankle angle was used to track the location on the Force–Length curve and showed the MG operates on the curve’s ascending limb. Muscle force changes predicted from the F-L curve going from dorsi- to plantarflexion was less than that experimentally observed suggesting other determinants of force changes with ankle position.

## Introduction

Dynamic studies of isometric and plantarflexion contraction in skeletal muscle using cine-MRI or velocity-encoded phase contrast magnetic resonance imaging (VE-PC MRI) have revealed several aspects of muscle deformation that require further exploration^1,2^. The muscle force–length (F-L) relationship is well established and describes the dependence of the steady-state isometric force of a muscle (fiber, or sarcomere) as a function of muscle (fiber, sarcomere) length and it has been explained by the ‘sliding filament’ theory^3–5^. In this theory, the maximal isometric force of a sarcomere is determined by the amount of overlap between the contractile filaments, actin, and myosin^4^. At short lengths, force increases as sarcomere length increases (ascending slope), reaches a plateau at intermediate lengths (optimal length for maximum force production), followed by a decrease in force as sarcomere length increases (descending slope) at long muscle lengths. Muscle fiber architecture clearly influences force production. A study of this structure–function relationship in-vivo will reveal aspects of force production that can be used to understand muscle physiology and to develop optimal exercise paradigms for rehabilitation or to maximize athletic performance^6–8^. The medial gastrocnemius (MG) force–length relationship can be altered by changing the knee joint position, ankle joint position, or both. The gastrocnemius muscle is biarticular and spans both the knee and ankle joints; thus, changes in one or both joint angles impact the resting MG muscle fiber architecture and consequently, the force produced by the MG. Prior studies have used electromyography (EMG) and ultrasound (US) to study muscle isometric plantarflexion force, activation, and muscle fiber architecture changes in the MG for combinations of knee flexion and ankle angles^9–12^. These studies showed that while there were significant differences in fascicle length of the MG at rest for the different knee/ankle positions, these differences in length were not seen at a maximal isometric plantar flexion contraction (100% Maximum Voluntary Contraction (MVC)). Further, the EMG activity of the biarticular MG at MVC decreased at a pronounced flexed knee-joint position despite the fact there were no differences in MG fascicle length^10^. The authors concluded that the decrease in EMG activity of the MG at pronounced knee flexed positions is due to a critical force–length potential of all three muscles of the triceps surae^10^.

The force produced by contracting muscle fibers is transmitted to bones via two passive structures: the aponeurosis and the tendon. These tendinous tissues play an important role as series-elastic-components and can store elastic energy during the movement^1,13,14^. However, it should be noted that some studies have identified other potential ‘passive’ load bearing structures such as neurovascular tracts^15,16^. The VE-PC MRI technique showed that the strain was heterogeneous with the deep and superficial aponeurosis of the MG exhibiting positive and negative strains along the muscle length that was hypothesized to be linked to the distribution and orientation of the forces generated by the muscle fibers^1^. The heterogeneity of fiber length and pennation angle in the proximo- distal direction of the MG may cause nonuniformity of fiber shortening with corresponding changes in regional aponeurosis strain. It is thus likely that, since varying the ankle angle leadings to different muscle fiber architecture, it will not only affect muscle fiber strain but also aponeuroses strains. VE-PC MRI imaging has been successfully implemented to study muscle kinematics under different contraction paradigms^1,2,17,18^ (details of the VE-PC method in Supplemental Information). The current study based on the VE-PC technique for MR strain mapping extends the work by Karakuzu et al.^15^. It focuses on determining the strain in the MG muscle fiber and in its deep and superficial aponeuroses at different ankle angles (dorsiflexed and plantarflexed-low and high angles) and under different submaximal effort (25% and 50%MVC). Earlier work using dynamic MRI identified the muscle fiber direction by the fascicles on water suppressed images^2^. The fascicles are visualized as higher intensity on the water suppressed images due to the presence of fat adjacent to the fascicles. However, the fascicles are not always visualized consistently since there is little or no fat adjacent to the fascicles in younger participants. The current paper explores an alternate way to extract regional muscle fiber direction using diffusion tensor imaging (DTI) without the additional complexity of fiber tractography. Of relevance to the current paper, DTI provides the direction of muscle fibers at the voxel level and this feature is used in the current paper to extract the average fiber direction in regions-of-interest of the MG.

Prior studies did not measure muscle fiber strain and aponeurosis strain at varying ankle angles^9–12^. These studies established that the dorsiflexed ankle position produced the highest torque compared to the plantarflexed ankle positions and that fiber architecture was not significantly different between different knee/ankle angle positions at MVC while significant differences in fiber architecture were present at rest. The explanation advanced was that a lower limit of fiber length is reached below which a neural inhibition may limit further contraction. However, at sub-maximal activity levels, this lower limit of fiber length may not be reached. While studies at MVC provide physiological insights, it should be noted that muscular properties during submaximal activity cannot be accurately extrapolated by scaling of force to the muscle properties obtained at maximal activation^19^. Sub-maximal levels of activity are relevant since routine activities like gait are performed at this level. The purpose of the current study is to measure muscle fiber length, pennation angle and strain and aponeurosis strain at varying ankle angles at two sub-maximal force levels. The hypotheses are that, in the MG, at sub-maximal activity levels (i) fiber length and pennation angles differences across ankle angles will persist, (ii) MG fiber strain will be lowest in the dorsiflexed ankle position while producing the largest force, (iii) highest departure from linearity of muscle fiber strains with %MVC effort will occur in the plantarflexed position since the fiber length is closer to its ‘lower limit’ in this position, and (iv) MG aponeurosis strain patterns will vary with the ankle angle reflecting the influence of fiber architecture.

## Methods

### Participants

The study was approved by the Medical Research Ethics Board of University of California at San Diego (UCSD) and conformed to the standards in the Declaration of Helsinki on the use of human participants in research. All participants were included in this study after obtaining informed consent. Six healthy, moderately active, male participants were examined in this study, age: 33.2 ± 16.3 yrs. (range 24–66 yrs.), height: 172.5 ± 7.0 cm (range 163–180 cm), mass: 73.3 ± 6.5 kg (range 63–82 kg). Participants were excluded if they were involved in vigorous physical training at the level of competitive athletics for the previous 3 months and were also asked to refrain from strenuous activities/ exercise a few days prior to the imaging study. Estimates of the number of participants was based on an initial measurement of MVC at the dorsiflexed and plantarflexed ankle positions on a few participants; this power calculation yielded 3 participants to detect paired differences between MVC at the dorsiflexed and plantarflexed ankle positions at 80% power and a level of significance of 5%. Since the effect size between the dorsiflexed and plantarflexed ankle positions at MVC is anticipated to be the highest, the number of participants was doubled to detect smaller changes in strain and normalized strain between the three ankle angles at sub-maximal activity levels.

### MR imaging

MR imaging was performed on a 1.5 Tesla MR scanner (Signa HDx, GE Medical Systems, Milwaukee, WI) with a body coil and an 8-Ch cardiac coil; the body coil was only used for the large FOV (30 cm) imaging required to cover the lower leg and foot to obtain the ankle angle while all other imaging was performed with the cardiac coil. Imaging was performed with the participant lying supine, feet first, with the dominant leg secured in a foot pedal fixture. Supplemental Information and Supplemental Fig. F1a provides details of the patient setup, feedback for consistent contractions and the triggering and participant feedback. Supplemental Fig. F1b provides a closeup view of a participant in the foot pedal setup. The foot pedal fixture allowed for the foot to be positioned at three nominal ankle angles: plantarflexion high (***PH***) − 40°, plantarflexion low (***PL***) − 25°, and. dorsiflexion (***D***) 5°. The ankle was imaged at two plantarflexed angles (PH and PL) and one dorsiflexed (D) ankle angle with the PL ankle angle was close to the relaxed position of the foot. A large FOV image that included the ankle was collected at each foot position using the body coil to verify/estimate the ankle angle. High-resolution water saturated fast-spin echo (echo time (TE): 12.9 ms, repetition time (TR): 925 ms, Echo Train Length (ETL): 7, signal averages (NEX): 4, slice thickness/gap: 3/0 mm, field of view FOV: 30 × 22.5 cm^2^, matrix: 512 × 384) oblique sagittal slices of the calf muscle were collected where water signal is suppressed while the fascicles (fat) appear hyperintense. The slice with greatest fascicle visibility (oblique sagittal slice) was selected for Velocity-Encoded Phase Contrast (VE-PC) imaging (*SS* with more than 24 years of experience with VE-PC imaging of the plantarflexors selected the slice for dynamic imaging). The location and orientation of the oblique sagittal slice for VE-PC imaging was determined from a set of localizer images and multiple oblique slices. The slice was chosen to be oriented approximately perpendicular to the planes of the aponeuroses in the thickest region of the MG muscle; this has been verified in several prior studies to best represent a section in the plane of the muscle fascicles^20,21^. Supplemental Video V1 shows the 3D anatomical volume generated from the FSE images to visualize the anatomical context of the VE-PC slice. The dynamic gated VE-PC images were collected for a single oblique sagittal slice (TE: 7.7 ms, TR: 16.4 ms, signal averages (NEX): 2, flip angle (FA): 20°, slice thickness: 5 mm, field of view FOV: 30 × 22.5 cm, partial-phase FOV: 0.55, matrix: 256 × 192 (in-plane resolution of 1.172 mm (PE: 225/192) × 1.172 mm (Read-Out (RO): 300/256)) , gated 22 phases, 3-direction velocity encoding with *venc*: 10 cm/s, 53 repetitions [192 (phase encode lines) × 0.55 (partial FOV) × 2 (NEX)/4 (views per segment) = 53]) of 3 s isometric contraction cycles with a total scan time 2 min and 39 s. Supplemental Information provides details of the selection of the *venc* value for the muscle imaging protocol.

Force exerted by the participant during isometric contraction was detected by a strain sensor embedded in the foot-plate. Participants were provided real-time visual feedback of the force generated superposed on the target force curve to facilitate consistent contractions. Supplemental Video V2 shows the target force curve (3 s duration sinusoidal half wave) superposed on the actual force exerted by the participant. The differentiated force signal acted as the trigger for gated VE-PC image acquisition. As shown in the Supplemental Video V2, the trigger for any acquisition line occurs when the first derivative of the exerted force exceeded a pre-set positive threshold. This threshold value is adjustable and was set such that the trigger occurred at the rising part of the force curve. Diffusion Tensor Imaging (DTI) images were acquired anatomically and geometrically matched slice to the VE-PC slice, using a SE-EPI DTI (TE/TR = 63/2200 ms) sequence with one baseline image (b = 0) and with 32 diffusion gradient directions at a b-value of 400 s/mm^2^ (all at one average), spectral-spatial slice select RF pulses for fat suppression and phase encoding along the AP direction such that fat was shifted out of the medial gastrocnemius muscle. It should be noted that, since the acquisition was performed at 1.5 T, artifacts including that from susceptibility differences and from chemical shift (fat shifts) did not affect image quality significantly. The DTI slice was geometrically matched to the VE-PC slice and was at the same position, orientation, slice thickness and FOV (including 0.55 phase FOV) as the VE-PC slice. The DTI matrix was 128 × 96 to minimize artifacts and this resulted in an in-plane resolution of 2.34 mm × 2.34 mm. However, the scanner reconstructed all images to a size of 256 × 256 resulting in the DTI images matched to the size of the VE-PC images. DTI images were acquired for each ankle angle and corrected for eddy current artifacts, denoised^22^, and processed for the eigenvalues/ eigenvectors and fractional anisotropy.

It should be noted that history effects including fatigue from contractions at the prior ankle angle were not anticipated for the following reasons: (i) these were submaximal levels (25% and 50%MVC) and the dynamic acquisition was for 2:39 min only. Prior studies at submaximal contraction (25%MVC torque) sustained with the elbow flexor muscles required ~ 43 min to decrease MVC torque by 41%^23^. It can thus be safely interpolated that the 2:39 min duration in the current study will not significantly affect MVC torque. (ii) DTI of 7 min duration was acquired between the two %MVC efforts at any ankle angle. This ensured that there were no history effects from the prior MVC effort. (iii) Between the ankle angles, there was at least a ~ 10-min break to reposition the participant at the new ankle angle. Since history effects and fatigue were not anticipated, a constant order of ankle angle (***PH***, ***PL***,*** D***) was used in the protocol. Subjects found it more difficult to exert repeated contractions at the high plantarflexed ankle angle (***PH***), so this ankle angle was imaged first at the start of the study. Supplemental Fig. F2 summarizes the study and imaging protocol along with timings of each step.

### Force measurements

The foot pedal’s embedded strain sensor measurements were transmitted via optical fiber cable and recorded by a Data Acquisition device (National Instruments, TX, USA) connected to the computer. The ball of the foot rests on the embedded sensor and Supplemental Fig. F1a and b shows the details of the foot positioning. The foot pedal device was calibrated for force so the measured output was the force exerted on the sensor. Maximum Voluntary Contraction (MVC) was measured for each participant at each ankle angle as the best of three trials recorded prior to imaging: MVC_D_ = 271 ± 49N, MVC_PL_ = 140 ± 29N, MVC_PH_ = 66 ± 20 N (average over all 6 participants). The MVCs were significantly different between the three ankle angles: MVC_D-PL_ (*p* = 0.0012), MVC_PL-PH_ (*p* = 0.0003), and MVC_D-PH_ (*p* = 0.0012), where the subscripts are the two ankle angles compared in paired t-tests. VE-PC images were collected for submaximal contraction targets of 25% and 50%MVC.

### Muscle fiber identification

The deep and superficial aponeurosis of the MG muscle was manually identified (~ 30 s) on the VE-PC magnitude image while the rest of the muscle fiber identification process, described below, was automated. The in-house developed algorithm segmented the MG into three regions corresponding to top third (proximal), middle third (middle) and lower third (distal) of its total length. Each region was eroded (3 × 3 structuring element with corresponding dimensions of 7 mm × 7 mm) in order to avoid pixels close to the aponeurosis. This eroded region was filtered to remove all voxels with a Fractional Anisotropy less than 0.15 to exclude noise, fat, and other non-contractile voxels. Since the DTI eigenvectors are 180° indeterminate, the leading eigenvector at each voxel was aligned to point in the same quadrant. The average of the leading eigenvector of each voxel in the region was computed. A line with the average in-plane direction of each region was placed in each region’s center; the ends of this line were extended to intersect the superficial and deep aponeurosis of the MG. This line was designated as the representative fiber direction for the region; a ‘fiber direction’ was identified for each of the three regions. Fiber strain analyses was performed on this representative fiber direction. It should be noted that the out-plane component of the leading DTI eigenvector was small, confirming the orientation of the oblique sagittal image captured the MG fibers in-plane of the slice. The DTI out-plane components of the leading DTI eigenvector averaged over the six participants were small: 8.4%, 9.4% and 7.9% for dorsiflexion, plantar flexion (low and high) ankle-angle positions, respectively. The process of fiber identification for all ankle angles per participant took about three minutes including the manual identification of the aponeuroses of the MG.

### Fiber strains and pennation angle

Phase-contrast images were corrected for phase shading artifacts and denoised using a 2D anisotropic diffusion filter ^2^. The endpoints of the DTI-identified muscle fibers were tracked through each frame of the dynamic study using the velocity data. It should be noted that DTI data was obtained at rest only. The muscle fiber end points were identified on the DTI images obtained at rest that corresponded to the first frame of the dynamic VE-PC sequence (images were acquired in the contraction cycle with the first frame starting from rest). The muscle fiber in subsequent frames of the dynamic sequence was identified by tracking the fiber end points from the rest frame using the velocity data. Fiber angles were measured with respect to the y-axis of the image (SI direction). Changes in fiber angle were calculated from the initial angle of the fiber. Changes in fiber length were calculated with respect to the initial length and Lagrangian strains were computed (Supplemental Fig. F3). It should be noted that the Lagrangian strain is the change in length divided by the original length, while Eulerian strain is the strain divided by the instantaneous length. Cardiac and skeletal muscle deformations are best described by the Lagrangian approach where displacement and strain are described around a point/region in tissue as it moves through space and time^24^. Eulerian description on the other hand, focuses on displacement and strain information at several locations in muscle tissue, which may change as time elapses. As a result, it is not an optimal way to describe tissue deformation properties with time. In the current study, the Lagrangian strain was calculated from the difference between the original length of the fiber and the length of the fiber (the latter estimated from the fiber end points tracked through the dynamic cycle) at each temporal frame normalized to the original fiber length. The strain values at the peak of the contraction (peak of the force curve) are reported in the current study. The strain relative to the generated force adds information on the efficiency of force production, i.e., the amount of deformation required for unit increase in force. Towards this quantification, fiber strains normalized to force were also computed. Strain, changes in fiber length and angle used in the statistical analysis were computed at the peak of the force curve. The analysis of fiber strains and pennation angle is completely automated and was completed in less than 30 s per participant (Supplemental Fig. 3 gives the flow chart for image analysis).

### Aponeurosis analysis

The deep and superficial MG aponeuroses were manually identified on the first frame of the magnitude VE-PC image for each participant (Supplemental Fig. F3). Each aponeurosis was divided into 11 equal length segments starting from the proximal end of the tibia to the distal end of the MG and tracked through the frames of the dynamic study^1^. Care was taken to position the points in the signal bearing region (muscle) adjacent to the aponeurosis but not on the aponeurosis itself which has no signal intensity. Length and Lagrangian strains were calculated for each aponeurosis segment for all temporal frames for each ankle angle and %MVC. The identification of the aponeuroses was performed by *BC* and verified by *US* and *SS* (12 and 24 years of experience respectively). This step of the analysis including manual deep and superficial aponeurosis identification followed by automated analysis of segmental strain and visualization completed in about 3 min per participant.

### Analysis of sarcomere length and F-L curve

An exploratory analysis was also conducted to determine if the changes in MVC with ankle angle can be explained by the relative positions of the muscle (sarcomere) length on the F-L curve. To this end, the relative sarcomere length at different ankle angles was computed assuming a reference sarcomere length for the dorsiflexion ankle position. The reference value was chosen such that it was closer to the optimal sarcomere length for maximum force (on the F-L curve) since the dorsiflexor position generated the maximum force (compared to the other two ankle positions). A sarcomere length of 1.9 μm was assumed for the dorsiflexion ankle position; the choice of this reference sarcomere length in the D position was made to approximately follow the changes in MVC determined experimentally when going from D to PL to PH. For example, choosing a sarcomere length closer to the peak of the F-L curve (2.5 microns) resulted in much smaller changes in MVC as a function of the ankle angle than observed experimentally. Assuming a sarcomere length of 1.9 µm for the D position and with the measured fiber length, the number of sarcomeres was computed. As the number of sarcomeres is not expected to change with ankle angle, the same sarcomere number was used to calculate the sarcomere length for muscle fiber length at rest and at 50%MVC at all ankle angles.

### Statistical analyses

The outcome variables of the fiber analysis are: fiber strain, fiber strain normalized to force, fiber length, and pennation angle; the latter two in the rest and peak contraction frames. Normality of data was tested using both the Shapiro–Wilk test and visual inspection of Q-Q plots. Three-way repeated measures analysis of ANOVA showed that there were no significant differences with fiber location (in the three regions: proximal, middle and distal) in any parameter except in the pennation angle. The values were averaged for the three regions in order to decrease the number of independent variables. Fiber strain and normalized fiber strains were normally distributed and for these variables, changes between ankle angles, %MVC as well as potential interaction effects (ankle angle x %MVC), were assessed using two-way repeated measures ANOVAs and in case of significant ANOVA results for the factor ‘ankle angles’, Bonferroni-adjusted post-hoc analyses were performed. When interactions were present, simple main effects were also examined. Muscle fiber length and pennation angles at rest and peak contraction were not distributed normally, so non-parametric testing was used. For these four outcome variables, the related samples test with Friedman’s 2-way ANOVA by ranks with corrections for multiple comparisons was performed for ankle angle. For all tests, the level of significance was set at α = 0.05. Data are reported as mean (SD) for the variables that are normally distributed and as median (interquartile range, IQR) for those not normally distributed. The statistical analyses were carried out in SPSS for Mac OSX (SPSS 28.0.1.1, SPSS Inc., Chicago, IL, USA).

## Results

**Figure 1:**
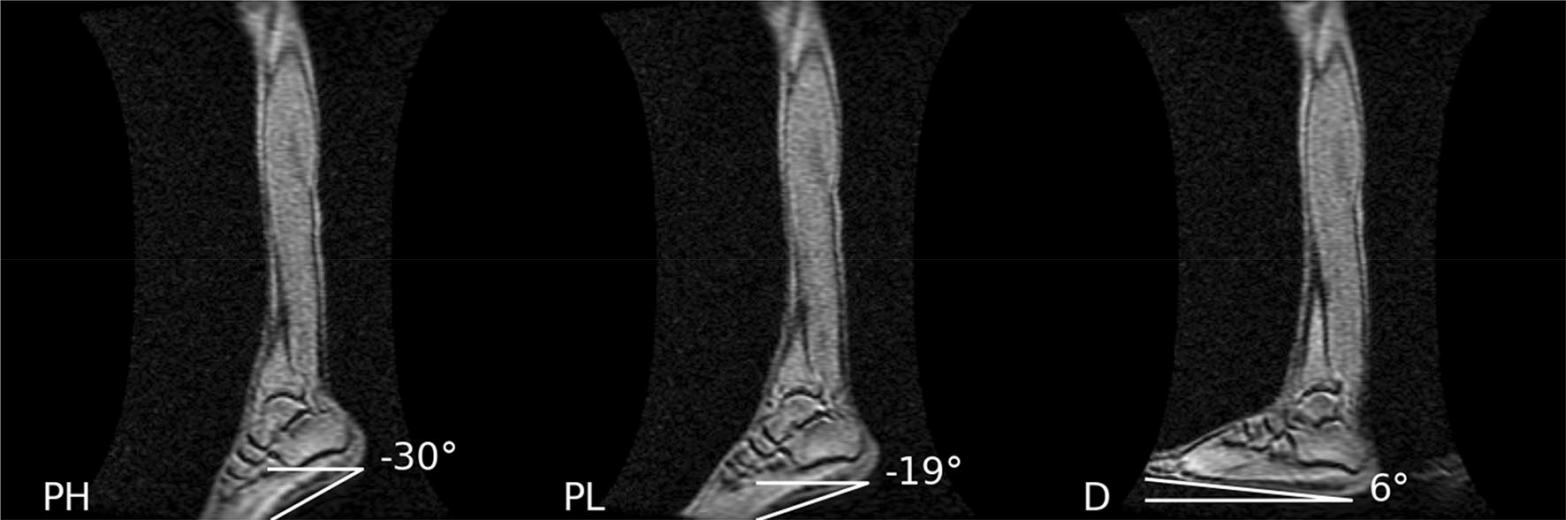
Large field of view (FOV) straight sagittal images using the body coil for one participant at the three ankle angles (left: plantarflexed -high, middle: plantarflexed -low, right: dorsiflexed ankle positions). The images document the actual ankle angle as shown here in the angle measurements at each ankle position. The solid white lines to identify the ankle angles were drawn as follows: both lines are started at the center of the heel. Then one line was horizontal to the x-axis and the other was parallel to the edge of the foot.

**Figure 2:**
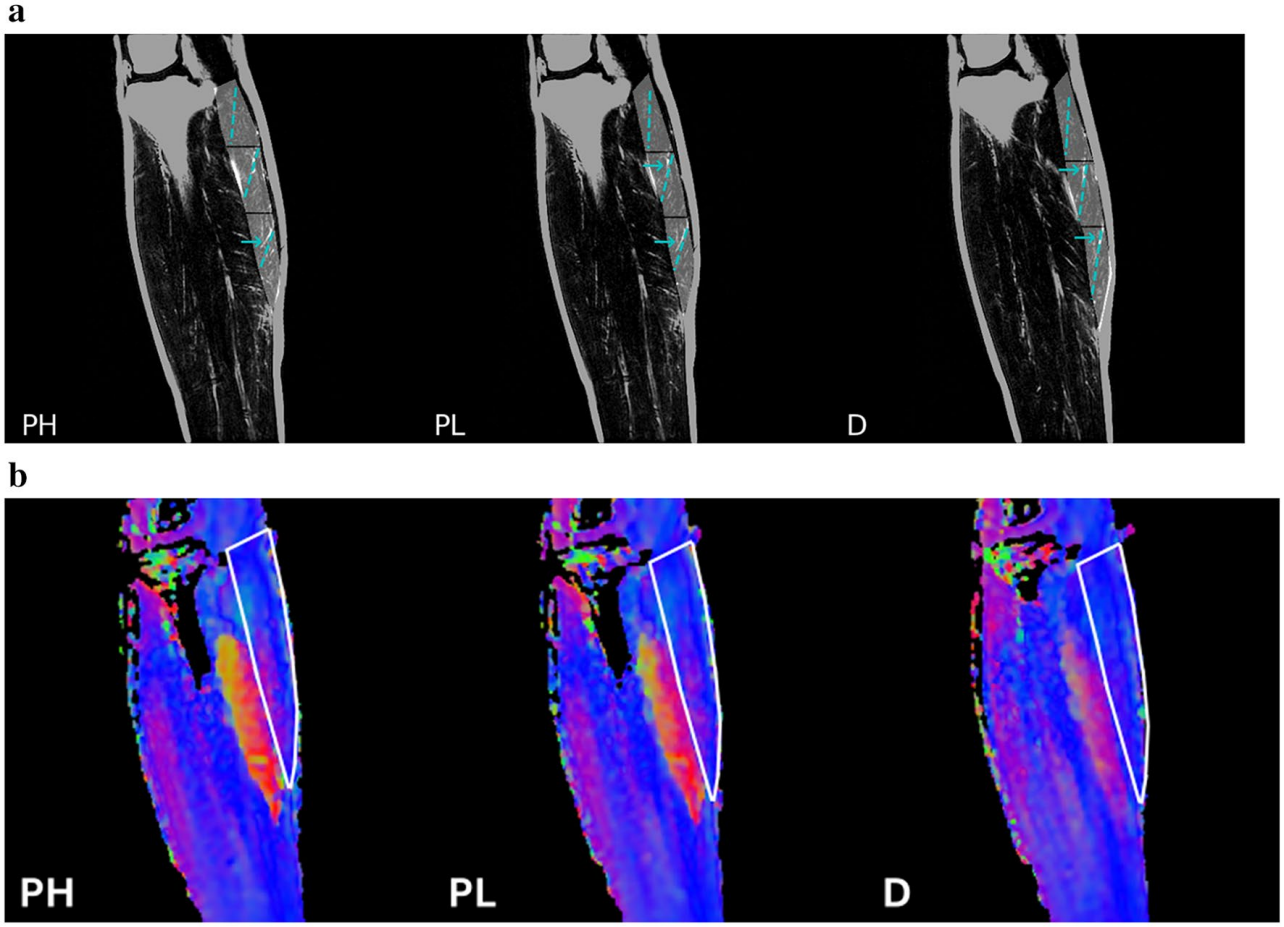
(**a**) From left to right: Images at plantarflexed high (PH), plantarflexed low (PL) and dorsiflexed (D) ankle angles. The fibers identified by the proposed method using the DTI leading eigenvector data are shown in blue dashed lines in the three regions of the muscle (proximal, middle and distal regions bounded by solid black lines between the regions and shaded in gray) superposed on the water saturated Fast Spin Echo images (muscle appears dark while the fascicles appear bright due to the presence of fat on these images). A few fascicles in the MG can be seen on the water saturated images and these are approximately aligned with the DTI derived fibers (identified by blue arrows). It can be seen that not many fascicles are visible in the MG while the proposed technique using DTI is effective in identifying the muscle fibers. The mean value of the leading eigenvector data from each region was used to calculate the mean orientation of fibers in that region shown by the dotted blue lines. (**b**) From left to right: Diffusion Images at plantarflexed high (PH), plantarflexed low (PL) and dorsiflexed (D) ankle angles. Colormaps of the lead eigenvector are generated as follows: x-component of the eigenvector determines the red, the y-component of the eigenvector determines the green while the z-component of the eigenvector determines the blue hue in each voxel. The colormap of the MG (outlined in white) shows predominantly proximo-distal with some mediolateral orientation of the fibers with the latter component decreasing going from the PH to D ankle angle.

Figure 1 shows the ankle angle measurements determined from large FOV straight sagittal slices. Figure 2a shows the fibers identified by the average of the leading eigenvector method for the proximal, middle, and distal regions of the MG for each %MVC and ankle angle; the fibers are superposed on high-resolution water saturated fast-spin echo images. The orientation of the fibers is as expected for the MG and follows the direction of fascicles. Region-specific fibers are extracted from the automatically segmented proximal, middle, and distal regions (Fig. 2a). Figure 2b is the colormap of the lead eigenvector of the DTI tensor that indicates the MG fibers are oriented predominantly in the proximal–distal direction with a smaller mediolateral component; the colormap is fairly uniform showing the consistent orientation of the MG fibers. Supplemental Video V3 shows the cine images of the fibers with the motion of the fiber end points tracked through the dynamic cycle at two %MVCs and three ankle angles. The white solid lines are the fibers identified from the diffusion tensor images and are representative for each region. Strain was calculated only for the fibers shown in this video and not for the entire region.

**Figure 3:**
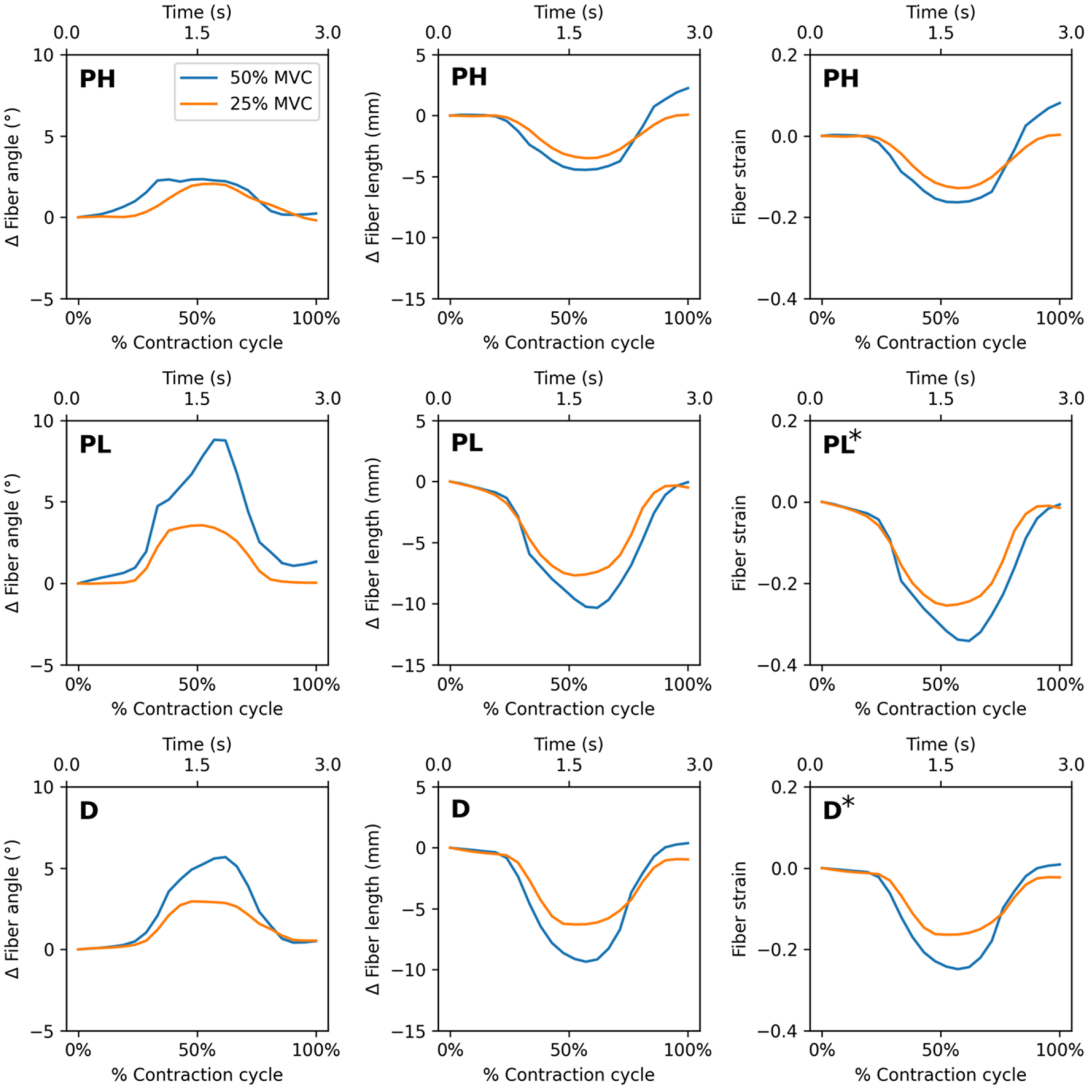
The variation, as a function of the dynamic cycle, of change in fiber angle from the initial frame, change in fiber length from the initial frame and fiber strain is shown for one participant (top row: plantarflexed high (PH) ankle position, middle row: plantarflexed low (PL) ankle position, bottom row: dorsiflexed (D) ankle position. The changes with %MVC is the least for the plantarflexed high position. The values at the peak of the contraction are reported in Tables 1 and 2 (averaged over the six participants). * indicates significant differences in fiber strain between 25 and 50%MVC (PL and D ankle angles only).

Figure 3 depicts the changes in fiber angle, length and strain as a function of the contraction cycle for one participant. Table 1 lists the mean and standard deviation of MVC, peak force, fiber strain, and normalized fiber strain for each foot position and %MVC. Table 2 lists the median and interquartile range of fiber architecture at rest and at peak contraction. The peak strain was significantly lower at the lower %MVC (*p* = 0.002) and while peak strain was the lowest in the dorsiflexed position, it was not significantly different from other two ankle angles (Table 1). Further, fiber strain changed significantly between 25 and 50%MVCs in the dorsiflexed (*p* = 0.004) and plantarflexed-low (*p* = 0.034) positions but not in the plantarflexed-high position (Table 1). As (ankle angle * force) interaction was significant for strain normalized to force, simple main effects are reported for strain normalized to force. At both 50%MVC and 25%MVC, strain normalized to force was significantly different between each pairwise combination of ankle angles (Table 1). At 50%MVC the p-values for the differences in normalized strain between ankle angles were the following: D-PL (*p* = 0.022), D-PH (*p* = 0.004), PL-PH (*p* = 0.012) and corresponding values at 25%MVC were *p* = 0.037, 0.012, 0.016 respectively. The absolute value of the normalized strain was lowest at the dorsiflexed position at 50%MVC while the highest normalized strain was at the plantarflexed position at 25%MVC (Table 1). Normalized strain at 50 and 25%MVC at each ankle angle showed that significant changes with %MVC was only seen in the PH ankle position (*p* = 0.029). The absolute value of normalized strain at 25%MVC was significantly higher than at 50%MVC for the PH ankle angle. Significant differences were seen in the resting fiber length between D-PH (*p* < 0.001) and D-PL (*p* = 0.023) and in resting pennation angle between D-PH (*p* = 0.029) while a trend was observed in D-PL (*p* = 0.059) and PL-PH (*p* = 0.059) (Table 2). Resting fiber length decreased and pennation angle increased going from D to PL (Table 2). Significant differences in fiber length at peak contraction were seen with force (*p* < 0.001) and with ankle angle: D-PH (*p* < 0.001) and D-PL (*p* < 0.001). Significant differences in pennation angle at peak contraction were seen with force (*p* < 0.001) and with ankle angle: D-PL (*p* = 0.029) and D-PH (*p* < 0.001). At peak contraction, fiber lengths decreased and pennation angles increased compared to corresponding values at rest.

**Table 1:** Muscle fiber strain and fiber strain normalized to force.

Ankle position	%MVC	MVC (N)	Peak force (N)	^1d^Peak strain	^1a^^,1b,1c,1d, 2a,2b,2c,2d^Strain/force (1/N)
D	50%	271.0 [48.7]	131.0 [31.1]	− 0.1545 [0.0656]	− 0.0013 [0.0007]
D	25%	271.0 [48.7]	68.5 [15.7]	− 0.1029 [0.0466]	− 0.0015 [0.0007]
PL	50%	140.2 [29.2]	71.9 [20.1]	− 0.2179 [0.0945]	− 0.0032 [0.0016]
PL	25%	140.2 [29.2]	36.2 [10.4]	− 0.1539 [0.0774]	− 0.0045 [0.0025]
PH	50%	66.4 [20.5]	31.6 [13.9]	− 0.2084 [0.0730]	− 0.0076[0.0036]
PH	25%	66.4 [20.5]	16.4 [7.4]	− 0.1742 [0.0694]	− 0.0123 [0.0061]

Ankle positions are dorsiflexion (D), plantarflexed-low (PL), plantarflexed-high (PH).

Standard deviation values are provided in square brackets beneath mean values.

^1a^50%MVC: Significant difference between ankle positions D and PL.

^1b^50%MVC: Significant difference between ankle positions PL and PH.

^1c^50%MVC: Significant difference between ankle positions D and PH.

^1d^D and PL: Significant difference between 25 and 50%-MVC.

^2a^25%MVC: Significant difference between ankle positions D and PL.

^2b^25%MVC: Significant difference between ankle positions PL and PH.

^2c^25%MVC: Significant difference between ankle positions D and PH.

^2d^PH ankle angle: Significant difference between 25 and 50%MVC.

**Table 2:** Muscle fiber architecture at rest and at peak contraction.

Ankle position	%MVC	^b,c^Rest angle (˚)	^a,c^^,d^Peak contr. angle (˚)	^a,c^Rest length (mm)	^a,c^^,d^Peak contr. length (mm)
D	50%	32.2 [1.5]	34.1 [3.3]	42.6 [13.6]	35.9 [11.4]
D	25%	32.2 [1.5]	33.4 [4.6]	42.6 [13.6]	38.8 [13.0]
PL	50%	33.3 [3.5]	34.6 [7.3]	34.5 [11.8]	28.2 [10.7]
PL	25%	33.3 [3.5]	33.2 [5.3]	34.5 [11.8]	32.0 [13.8]
PH	50%	35.1 [4.3]	37.3 [5.7]	29.0 [5.8]	23.0 [6.2]
PH	25%	35.1 [4.3]	36.6 [5.6]	29.0 [5.8]	25.2 [5.2]

Ankle positions are dorsiflexion (D), plantarflexed-low (PL), plantarflexed-high (PH) Interquartile range is provided in square brackets beneath median values.

*Contr.* contraction.

^a^Significant difference between ankle positions D and PL.

^b^Trend observed between ankle positions (D and PL), and (PL and PH).

^c^Significant difference between ankle positions D and PH.

^d^Significant difference between 25 and 50% of maximum voluntary contraction (MVC).

**Figure 4:**
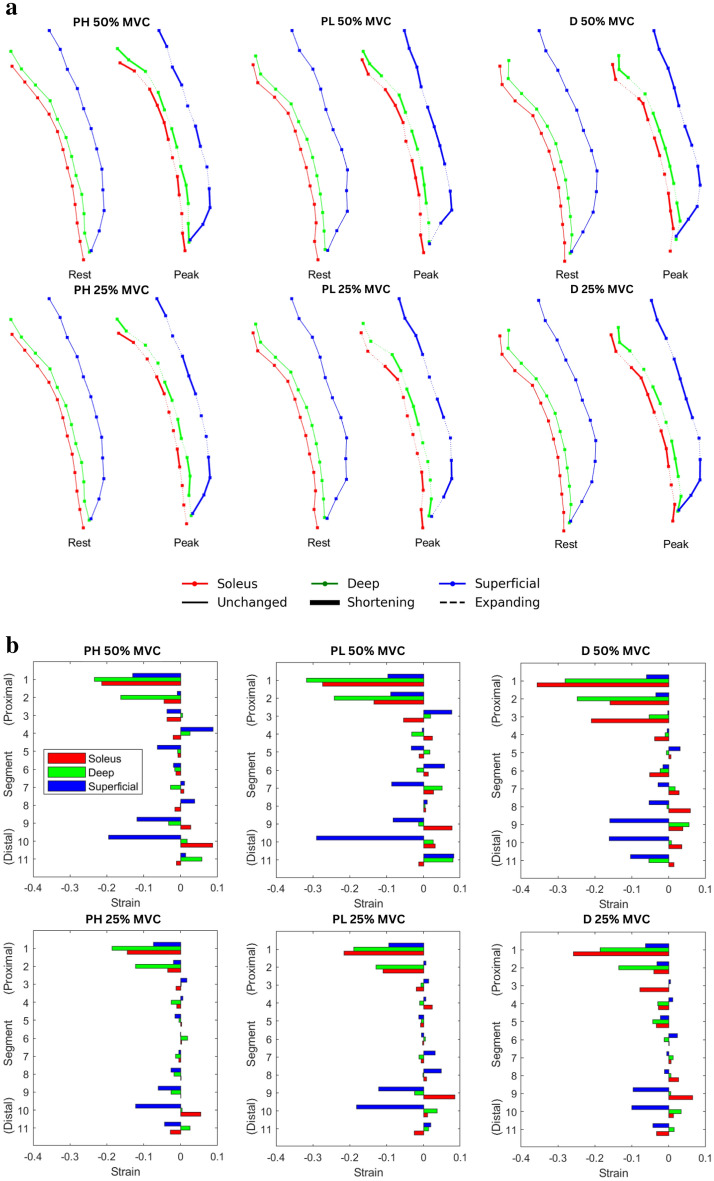
(**a**) The end points of the 11 segments are shown for the medial gastrocnemius deep (green) and superficial (blue) aponeuroses. The points on the soleus to track the aponeurosis are shown in red. The top row shows 50%MVC while the bottom row shows 25%MVC. The schematic is presented in pairs (at rest and at peak contractions) from left to right: plantarflexed-high (PH), plantarflexed-low (PL), and dorsiflexed (D) ankle angles. In the paired schematics, the segments at the moment of peak contraction are shown as shortening (thick solid line), expanding (dotted line) or unchanged (thin solid line) for the soleus aponeurosis (red), deep aponeurosis of the MG (green) and superficial aponeurosis of the MG (blue). (**b**) Plot of the segmental strain values extracted at peak of the dynamic contraction for 50%MVC (top row) and for 25%MVC (bottom row) for plantarflexed-high (PH), plantarflexed-low (PL), and dorsiflexion (D) ankle angle positions. Segment 1 is the proximal end while segment 11 is the distal end of the aponeuroses, the segments are arranged vertically starting from distal (lower end) to proximal end (top) so that it aligns with the segments shown in (**a**). Each segment has three bar plots corresponding to red (Soleus), green (deep aponeurosis), blue (superficial aponeurosis); there are eleven sets with three bar plots each corresponding to the eleven segments in (**a**). The close match of the segments tracked from the soleus and medial gastrocnemius sides of the distal aponeurosis (red and green, respectively) is a check of the internal consistency of the velocity-based tracking.

Figure 4a shows the schematic of the locations of the 11 points and line segments placed along the MG deep and superficial aponeuroses at rest and at peak contraction. It is to be noted that analysis was performed only on the segmental end points, thus intervening points within a segment cannot be displayed. Figure 4b is the column plot of the aponeurosis strain (estimated at peak force) for the two %MVC and the three ankle angles averaged over the six participants. Analysis of the strain comparing corresponding segments revealed no significant differences between the three ankle angles. Across all three ankle angles, aponeurosis strain was high at the distal and proximal regions of the muscle length and lowest in the middle where it was close to zero strain. The distal end of the deep aponeurosis showed small positive strains whereas the superficial aponeurosis revealed negative strains at the distal end. Further, for the superficial aponeurosis, the absolute value of strains at the distal segments were the highest among all the segments. The deep aponeurosis showed the highest negative strains in the proximal end, higher than strain values in the superficial aponeurosis. The motion of the segments along the aponeurosis during the contraction cycle is shown in Supplemental Video V4. The artifacts seen in the background in the phase encode direction in Supplemental Video V4 are ringing artifacts arising from gross motion; the artifact intensity was only a small fraction of the muscle tissue intensity. Dynamic MR images were inspected for artifacts from gross motion and excluded from further analysis if they were severe. Further, the video also shows that in two of the subjects, the points close to the aponeurosis in the distal most segment move well into the muscle at high velocities. This can potentially lead to errors but since it is confined to only one segment in two subjects, it is not anticipated that it will bias the overall conclusions.

**Figure 5:**
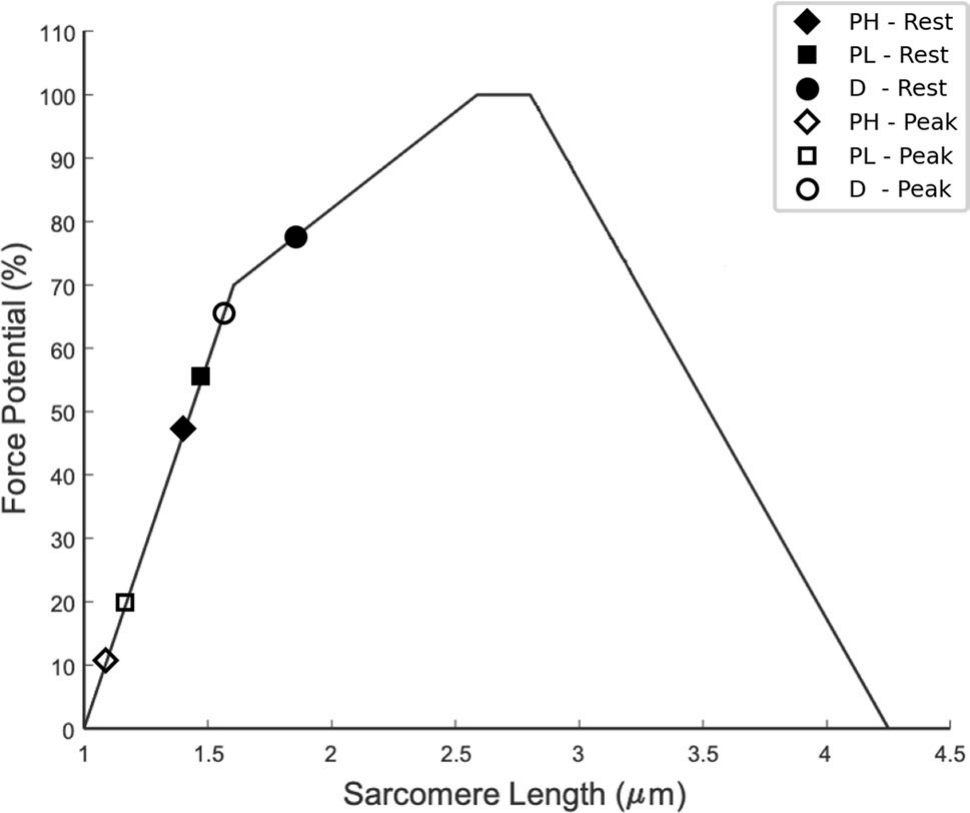
The Force- Length curve (solid line) is plotted based on data from Ref. 24. The black solid markers pertain to the sarcomere length calculated for the resting fiber length at each ankle angle (Plantar flexion, high: PH, Plantar flexion, low: PL, Dorsiflexion: D) while the black open markers are calculations made with lengths at peak contraction at 50%MVC (Table 3). All values were calculated using average values of fiber lengths for the six participants. A reference estimate of 1.9 microns for the sarcomere length in the dorsiflexed position was made in order to approximately follow the experimentally observed changes in force with ankle angle. The sarcomere number calculated at the dorsiflexed position was kept constant across the plantarflexed low and plantarflexed high positions.

The number of sarcomeres was calculated based on a reference sarcomere length of 1.9 microns for the D position and the corresponding measured fiber length at this ankle position at rest. As the number of sarcomeres is not expected to change with ankle angle, the same sarcomere number was used to calculate the sarcomere length at all ankle ankles at rest and at 50%MVC (Table 3). Figure 5 shows the sarcomere lengths calculated from muscle fiber lengths at rest (black-solid) and at 50%MVC (black-open) superposed on theoretical F-L relationship which was derived from data reported in^25,26^. The plot shows the MVC that can be attained for muscle fiber lengths measured at rest for the three ankle angles and provides a qualitative explanation for the decrease in MVC when the ankle angle changes from D to PL to PH.

**Table 3:** Muscle fiber length and sarcomere length at rest and at peak contraction.

Ankle position	Number of sarcomeres	Rest sarcomere length (μm)	Rest fiber length (mm)	Peak contr. sarcomere length (μm)	Peak contr. fiber length (mm)
D	24,526	1.9	46.6	1.6	39.8
PL	24,526	1.5	37.6	1.2	29.9
PH	24,526	1.4	33.2	1.1	26.6

Ankle positions are dorsiflexion (D), plantarflexed-low (PL), plantarflexed-high (PH).

*Contr.*: contraction.

## Discussion

The specific objectives of this study were to implement a diffusion tensor imaging (DTI) method to identify MG fibers and to map fiber strains using velocity-encoded phase contrast magnetic resonance imaging (VE-PC MRI) for submaximal isometric contraction with ankle angle held in dorsiflexion, plantarflexion-low, and plantarflexion-high angles. Prior work used fascicles that were manually identified on water saturated anatomical images but is limited in utility due to the difficulty of identifying fascicles; the method proposed here to identify fiber directions from the leading eigenvector of the DTI data is shown to be feasible.

The significant changes in MVC with ankle angle in isometric contractions seen in the current study have been reported in earlier studies^10^. The dependence of the MVC on the ankle angle is attributed to ankle dorsiflexion stretching the gastrocnemius and bringing it closer to its optimal length in the force–length curve, thereby enabling greater muscle force^10^. In all ankle positions, as anticipated, fiber length decreased and pennation angle increased at peak contraction compared to the values at rest. The current study confirms US studies that reported fiber lengths and pennation angles to be significantly different between ankle positions at rest^10^. However, the current study also shows that significant changes in fiber length and pennation angle with ankle angle (between D-PL and D-PH) persisted at peak contraction contrary to US studies that found no significant differences at peak force between low knee flexion/dorsiflexed ankle angle and high knee flexion/plantarflexed ankle angle^10^. This may reflect the fact that US studies were at 100%MVC whereas the current study was at submaximal isometric contractions. However, it should be noted that a direct comparison between Ref.^10^ and the current study is not possible since the former study changed both the knee flexion and ankle angle while only the ankle angle is changed in the current study. The current results confirm earlier studies that highlighted the fact that muscular properties during submaximal activity cannot be determined by scaling of force to the muscle properties obtained at maximal activation^19^. Further, Heroux et. al reported using ultrasound imaging during ramped submaximal contractions (0–25%MVC) that differences in fiber length and pennation angles persisted between neutral and plantarflexed ankle angles, corroborating the findings of the current study^27^.

EMG activity of the MG has been reported to decrease during MVC for knee flexed positions and more plantarflexed ankle angles^10^. The main mechanism postulated in Ref. ^10^ for decreasing EMG activity and impairment in neuromuscular transmission-propagation at short muscle lengths is a neural inhibition. The latter is triggered as the muscle reaches a critical shortened length at which, due to the F-L relationship, the torque output cannot be increased even if the muscle is fully activated. Kennedy and Cresswell concluded from single motor-unit activity and EMG measurements on the MG at two knee angles that motor units in the shortened muscle maybe influenced by a specific inhibition of motor units having shortened, non-optimal fascicle lengths and are thus are either incapable or have a reduced contribution to plantar flexor torque^28^. In the current study, MG fiber strain is higher at the PH ankle position compared to the D ankle position while MVC at D ankle angle was higher than the MVC at PH ankle angle. This points to the fact that the MG is inefficient in producing force in the plantarflexed ankle position. The MG did not show decreased strains at the PH ankle angle when going from 25 to 50%MVC indicating that it is not active insufficient; this finding is supported by prior work that showed that the gastrocnemii are not active insufficient in a knee fully extended position^29^.

It may also be likely that the higher strain in the PH ankle position does not lead to a greater force production due to a potential decrease in the efficiency of lateral transmission pathways. Support for this comes from an earlier study where a measurement of the strain tensor at three ankle angles showed shear strains to be the highest in the dorsiflexed position^30^. Prior MR studies found a significant positive correlation of shear strain to force in a cohort of young and old subjects or force loss due to unloading^31,32^. Further, one study reported intramuscular pressure (IMP) and EMG during isometric dorsiflexion MVC and ramp contractions at different ankle positions^33^. IMP was significantly correlated to the ankle torque during ramp contractions at each ankle position tested. However, the IMP did not reflect the change in the ankle torque which changed significantly with ankle positions. Similar to the IMP study, the current study also showed that compressive strains at each ankle angle did not reflect the change in MVC at different ankle angles.

Fiber strains in the MG were lower (though it did not reach significance) in the D ankle position compared to the PH and PL position and significantly higher at 50%MVC than at 25%MVC in the D and PL positions. The increased strain at higher %MVC in the D and PL ankle positions is understandable as increased contraction is required to generate the higher forces. In contrast to D and PL, the observation that the PH ankle position did not show significant change in fiber strain with %MVC may indicate that the MG may be approaching the critical length where further contraction becomes more difficult. It is surprising that the fiber strains are lower in the D ankle position since in this ankle position, higher force is generated. It would be expected that the higher force in the D ankle angle will be accompanied by larger strains; the implication of the lower strains is that the D ankle angle position is ideal for force generation in that even small contractions (strains) are sufficient to generate large forces. Further, other factors that could potentially contribute to the high force generation capability at the D ankle position may include non-muscular connections between MG and the soleus not only through the Achilles tendon but also through neurovascular tracts and fortified anatomic structures traversing deep GM and superficial soleus aponeuroses. Fiber strains normalized to force showed significant differences with ankle positions at both force levels. In comparing ankle positions, the D ankle position showed significantly lower normalized strains at both %MVCs than the PL and PH ankle positions; highlighting the fact that the D ankle position is at the optimum position for force production followed by PL and the least optimal for the PH ankle position. This could be physiologically relevant, e.g., during gait and quiet balanced standing. Smaller strains (contractions) are required to generate the same force at the dorsiflexed position than in the other two positions. Further, the absolute value of the normalized fiber strain was significantly higher at 25%MVC than at 50%MVC for the plantarflexed-high position. The latter finding is a reflection of strain not increasing significantly with %MVC in the PH ankle position. It is likely that compared to the D and PL positions, the contribution of the soleus to the total force increases in the PH ankle position at higher %MVCs.

In addition to the above factors to explain the experimentally observed strains at the different ankle angles, different passive strain of the Achilles Tendon (AT) at the three ankle angles can also be a contributor. The passive strain of the AT in the PL position is close to zero (close to the rest length). In the PH ankle position, the AT might show negative strain values (length of the AT lower than the rest length). On the other hand, the passive strain in the D ankle position would show positive values (passive forces are transmitted to the AT). Similar changes in the generated force of the triceps surae muscles in the three investigated foot positions, would therefore introduce different AT elongation and thus different muscle fiber strain values.

In terms of the aponeurosis strains during isometric contraction, ultrasound studies showed that the superficial and deep aponeuroses of medial gastrocnemius (MG) are uniformly stretched along their lengths in opposite directions; the superficial aponeurosis is stretched distally, whereas the deep aponeurosis is stretched proximally^34^. However, the MRI study by Kinugasa *et al.* revealed heterogeneous aponeurosis strain patterns: positive strain occurred at proximal and distal ends of the deep aponeurosis and in the proximal region of the superficial aponeurosis while negative strain was observed in the middle region of the deep aponeurosis and in the distal region of the superficial aponeurosis^1^. Similar patterns of aponeurosis heterogeneity are also seen in the current study as in the earlier MR paper^1^ if it is noted that the proximal most position of the current study is the third segment of the study by Kinugasa et al. Contrary to the hypothesis, the strain patterns did not show significant differences between the three ankle positions.

The length of sarcomeres that are arranged in series within a striated muscle fiber is one of the most important determinants of muscle force^25^. As the ankle angle changes, the MG fibers and sarcomeres change length, which shifts the sarcomeres’ positions on the force–length curve and affects the force-generating capacity of the muscle. It should be noted that the current study does not measure sarcomere length directly at any of the ankle angles but given the highest value of force at dorsiflexion position, this ankle position was assumed to closer to the optimum sarcomere length than the PL or PH positions. Further, once the reference sarcomere length for the dorsiflexion ankle angle position is chosen close to the optimum sarcomere length of the F-L curve, the results shows that the sarcomere lengths of the MG at the other two ankle angles are positioned on the ascending limb of the F-L curve. Similar observations were made from ultrasound and dynamometry studies where it was shown that soleus acts on the ascending limb during active contractions^35^. The choice of the reference sarcomere length at 1.9 μm for the D position was dictated by considerations that the calculated sarcomere lengths at other ankle angles resulted in force decreases on the F-L curve close to that observed experimentally. Based on the F-L curve, the choice of the optimal sarcomere length of 2.65 μm (generates maximum force) severely underestimated the observed force decrease with ankle angle. In fact, even the reference value of 1.9 μm underestimates the force decreases with ankle angles. This potentially indicates that there may be other determinants of the observed force changes with ankle angle beyond changes in the sarcomere length. It should also be noted that while an isometric contraction implies no change in muscle length, it does not mean imply an unchanged muscle fiber length. This has been reported in prior work that noted fiber level strain heterogeneities along the muscle length as well as along the muscle fiber^15,36^. It is possible that the force generation potential is determined by an average of the rest and peak sarcomere length than just the rest sarcomere length. While the above analysis attributes all the force loss with ankle angle to the MG, it should be noted that the force measured at the tendon is the sum of forces generated by the soleus and gastrocnemii muscle. The ankle angle affects the fiber length and pennation of all three plantarflexor muscles which in turn will affect their force generating capacity^37^. However, some evidence that the ankle angle affects the MG more than it does the soleus comes from EMG activity of the triceps surae muscles measured under a combination of knee and ankle flexion at MVC^10^; this latter study showed that there are significant differences in EMG across the different knee/ankle positions in the MG but not any in the soleus. The current analysis attributes all the force decrease at plantarflexed positions to the MG while acknowledging that some of it may well come from decrease in fiber length of the soleus as well. However, loss of soleus force with plantarflexed ankle angle may be the reason for the loss of force being much larger than would be predicted if the reference point was placed close to the optimum sarcomere length.

The limitations to the current study including the small cohort. However, sample size was informed by power calculations based on MVC differences between the different ankle angles. Further, it should be noted that the repeated measures ANOVA has higher statistical power since the variability in the participant population is taken into account and statistically significant differences were identified even in a small cohort. However, a larger cohort may enable one to discern regional differences in muscle fiber strain and normalized fiber strain. The force produced during isometric contraction is the force at the Achilles Tendon (AT) while the force reported in the current paper was measured at the ball of the foot. The AT force can be calculated by multiplying the measured force by the ratio of the distance from the fulcrum to the sensor (fixed distance) to the moment arm of the AT. Since the moment arm is known to increase with ankle angle plantarflexion, this will result in larger differences in MVC between the different ankle angles. Potentially it will lead to bigger size effects with ankle angle in MVC and force normalized strain but this will not change any of the significant differences reported in the current study. Further, it is acknowledged that moment arm changes during contraction were not accounted for. However, since sub-maximal MVCs were studied these moment arm changes may be smaller than reported at MVC^38^. Muscle fibers traverse a 3Dal space while the current study was in 2D. However, care was taken to position the oblique sagittal slice such that the fibers of the MG lay in the plane of the image; this was subsequently verified by the DTI based analysis. However, a 3D sequence is required to map fiber strains in all the calf muscles including the soleus and the plantaris muscle at different ankle angles and %MVC. The maximum %MVC possible for the studies was limited by the ~ 53 contractions to complete one acquisition. It is not possible to perform ~ 53 contractions at MVC. The level of 50%MVC was chosen based on prior experience as achievable by most subjects at the three ankle angles. In summary, the current paper focuses on muscle fiber and aponeurosis strains obtained at two levels of submaximal isometric contractions with the ankle angle varied from dorsiflexion to plantarflexed-low to plantarflexed-high ankle angles. The dorsiflexed ankle position generated significantly greater force while exhibiting significantly lower normalized strains than the two plantarflexed positions. The sarcomere lengths at rest and at peak force were calculated assuming a reference optimal sarcomere length at the dorsiflexed ankle angle and the MG was identified to be working in the ascending limb of the F-L curve. The latter analysis also revealed that there may be other determinants to force changes with ankle position in addition to changes in sarcomere length of the MG.

### Supplementary Information

Supplementary Information 1.

Supplementary Video 1.

Supplementary Video 2.

Supplementary Video 3.

Supplementary Video 4.

## Data Availability

The datasets analyzed during the current study are available from the corresponding author upon reasonable request.
