# Supplementary material for: Effect of different ankle joint positions on medial gastrocnemius muscle fiber strains during isometric plantarflexion
Source: Sci Rep. 2023 Sep 11;13:14986. doi: 10.1038/s41598-023-41127-z (PMC10495375; doi:10.1038/s41598-023-41127-z)
Supplement: Supplementary file 1 — Supplementary Information 1. [file 41598_2023_41127_MOESM1_ESM.docx]

**Supplemental Information**

**Effect of Different Ankle Joint Positions on Medial Gastrocnemius Muscle Fiber Strains During Isometric Plantarflexion.**

Brandon T Cunnane MS , Usha Sinha PhD , Vadim Malis PhD , Ryan D Hernandez MS, Edward Smitaman MD, Shantanu Sinha PhD

**Dynamic Imaging Setup**

The foot-pedal was mounted on a flat acrylic bed (Supplemental Figure F1a and Supplemental Figure F1b). The subject’s leg was immobilized by an adjustable strap anchored to the bed - this immobilization reduced any gross subject motion. An additional strap over the foot pedal device ensured that the base plate of the foot pedal did not move. Further, the base plate of the foot pedal was prevented from moving in the longitudinal direction by an additional plate that tightly locked into the baseplate and the edge of the bed. The center of rotation of the ankle was aligned with the center of rotation of the foot-pedal bracket with adjustable spacers placed below the leg for small adjustments in the vertical direction. The ball of the foot rested against the plate. With the force sensor. The latter plate could be anchored at the three ankle angles using pins inserted into corresponding holes between the arm carrying the foot plate and the outside frame. A long Velcro strap was wrapped around the foot pedal base on one end and hand-held by the participant at the other end to stabilize the participant during isometric concentration. The cardiac coil was placed over the foot pedal and over the calf muscle (shown on the right photograph of Figure F1b). A large restraint anchored to the table was the placed over the cardiac coil to further ensure that there was no gross motion (large restraint not shown in Figure F1b for clarity of underlying arrangement). The VE-PC images were examined for gross motion artifacts and the restraints were re-adjusted in the event of artifacts.

During isometric contraction, the transducer detected the force exerted against the foot pedal and subsequently this was converted to a voltage by a spectrometer (Fiberscan, Luna Innovations, Roanoke, VA). This voltage was used to trigger the MR image acquisition using custom built software developed in LabView (version 14.0.1.4008. National Instruments Inc., Austin, TX). In addition to serving as a trigger for the MR acquisition, the pressure transducer voltage output was recorded at a sampling rate of 200 Hz and later converted into units of force (N) based on a calibration of the system using disc weights.

The participant was provided with real-time visual feedback of the actual force generated by the participant which was superposed on the target force curve; this feedback facilitated consistent contractions. A sinusoidal waveform was used for the target curve with a half-wave played out during the 3 seconds period of contraction. Supplemental Video V2 shows the target force curve superposed on the actual force exerted by a participant. The image of the target force curve along with the force produced by the participant was projected onto a screen placed in the scanner room to provide real-time feedback on the contractions (Supplemental Figure F1). Also shown in the Supplemental Video V2 is the trigger for an acquisition, the latter occurs when the first derivative of the exerted force exceeds a pre-set positive threshold. This threshold value is adjustable and was set such that the trigger occurred at the rising part of the force curve (green vertical line in the Supplemental Video V2).


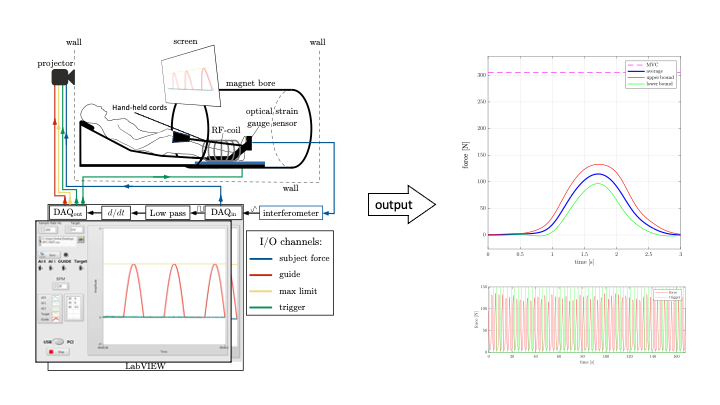


**Supplemental Figure F1a:** *Left top panel***:** Participant setup with dominant leg in the foot pedal device, centered in a cardiac flex coil (labeled RF coil), with visual feedback projected onto the screen for the subject to follow. Pressure against the carbon-fiber plate in the foot pedal was detected by the transducer and converted into voltage and then converted into measurements of force. Participant holds with both hands cords attached to a long Velcro strap that wraps around the back of the foot pedal device. *Left bottom panel:* The foot pedal output was processed to generate a trigger to synchronize with the MR acquisition and also displayed to the subject on a screen. *Right top panel:* The force curve averaged over ~53 contractions (required to acquire the MR images) is shown along with upper and lower boundaries of the force curve. *Right lower panel:* Plot of the force curves for one VE-PC acquisition and the green vertical lines are the triggers. The foot positioning for plantarflexed low (PL)ankle angle is shown here, plantarflexed high (PH), and dorsiflexed (D) positions were obtained by adjusting the ankle angle of the foot. This figure has been modified from the original available at (<https://escholarship.org/uc/item/9x42v0wq>); permission to use the original figure was obtained from the thesis author, V Malis.


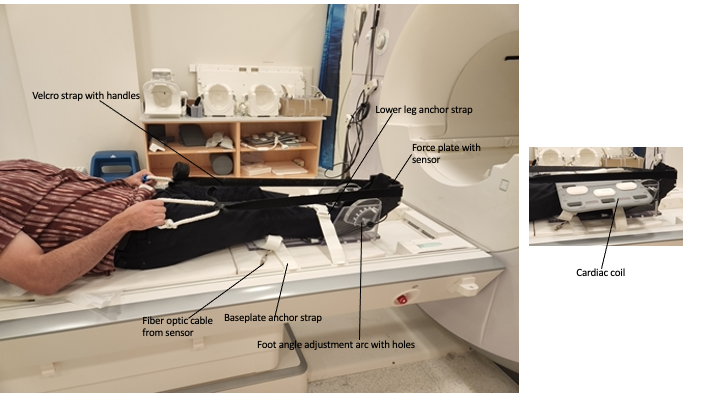


**Supplemental Figure F1b**: Typical patient setup shows the lower leg restrained by a white strap attached to the bed with a similar restraint for baseplate of the foot pedal device. The baseplate locked into another plate that fit snugly into the table space beyond the subject (not shown here). The participant was anchored by the handheld Velcro straps that passed behind the foot pedal endplate. The position of the foot (ankle angle) could be changed by rotating the force plate using the adjustment arc (peg can be adjusted in any of the holes in the arc). The cardiac coil is placed over the calf muscle and then a strap is placed over the coil attached to the table (strap not shown here for clarity).

**Velocity Encoded Phase Contrast (VE-PC) Imaging Pulse Sequence**

The VE-PC sequence has been extensively used to image and quantify blood flow; the velocity of blood can be as high as 200 cm/sec. On the other hand, muscle tissue velocities are much smaller and the VE-PC sequence has to be optimized to image velocities in the range of 1-5 cm/sec. Velocity is encoded by using a bipolar gradient; the strength of the latter gradient determines the maximum velocity that can be encoded before velocity aliasing occurs; the strength is quantified by the *venc* value (units of velocity). The *venc* value is selected such that it exceeds the maximum velocity (a rule of thumb is by 25%) that is anticipated in the study: choosing lower than the maximum velocity will lead to velocity aliasing while choosing a *venc* much larger leads to a lower sensitivity (smaller phase shifts for a given velocity change) and higher noise in the image. The optimal value of *venc* should be chosen such that it exceeds the maximum velocities anticipated; this requires some prior knowledge or adjustment for new contractions paradigms.

A *venc* value of 10 cm/s was chosen to cover the range of velocities that were experimentally observed in 30-65% MVC isometric platarflexion cycles performed over 3 second contraction cycles. This *venc* value covers the observed velocities in a range of subjects (25-70 years old) of both genders. A lower *venc* value would have yielded higher sensitivity to tissue velocities but this may cause aliasing at higher %MVCs or in participants with higher MVCs. In the current study, the MVC is highest in the dorsiflexed ankle angle but tissue velocities still did exceed 10 cm/sec.

In addition to the selection of the optimum *venc* value, the sequence is also optimized to reduce acquisition times. Reducing scan times is especially important for dynamic imaging as the subject has to perform repeated consistent contractions. Scan times were reduced in the imaging protocol used in the current study included partial phase FOV (since the leg in the oblique saggital) is not as wide as it is long, view sharing which allowed k-space lines to be shared between adjacent temporal frames. This allowed the dynamic imaging to be completed in 2 min 39 seconds with ~53 contractions.

**Study/ Imaging Protocol**

The study protocol is shown in Supplemental Figure F2. The time taken for each step is provided as well as for each imaging sequence. The image processing pipeline is shown in Supplemental Figure F3 and identifies manual and automated steps.


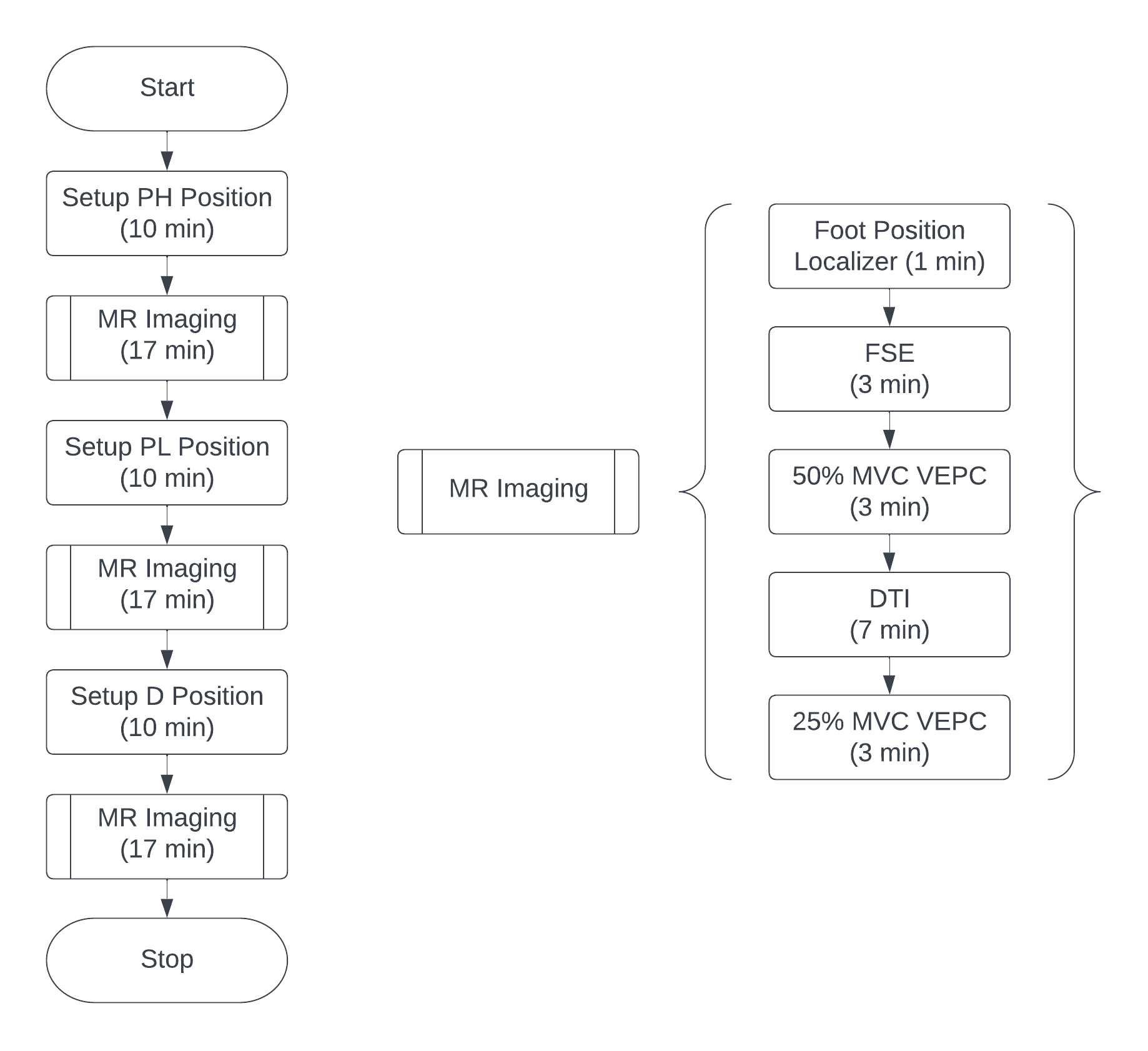


**Supplemental Figure F2:** The study protocol is detailed along with the time for the setup for the ankle angle position and MR imaging. The MR imaging protocol at each ankle angle is provided on the right flowchart.


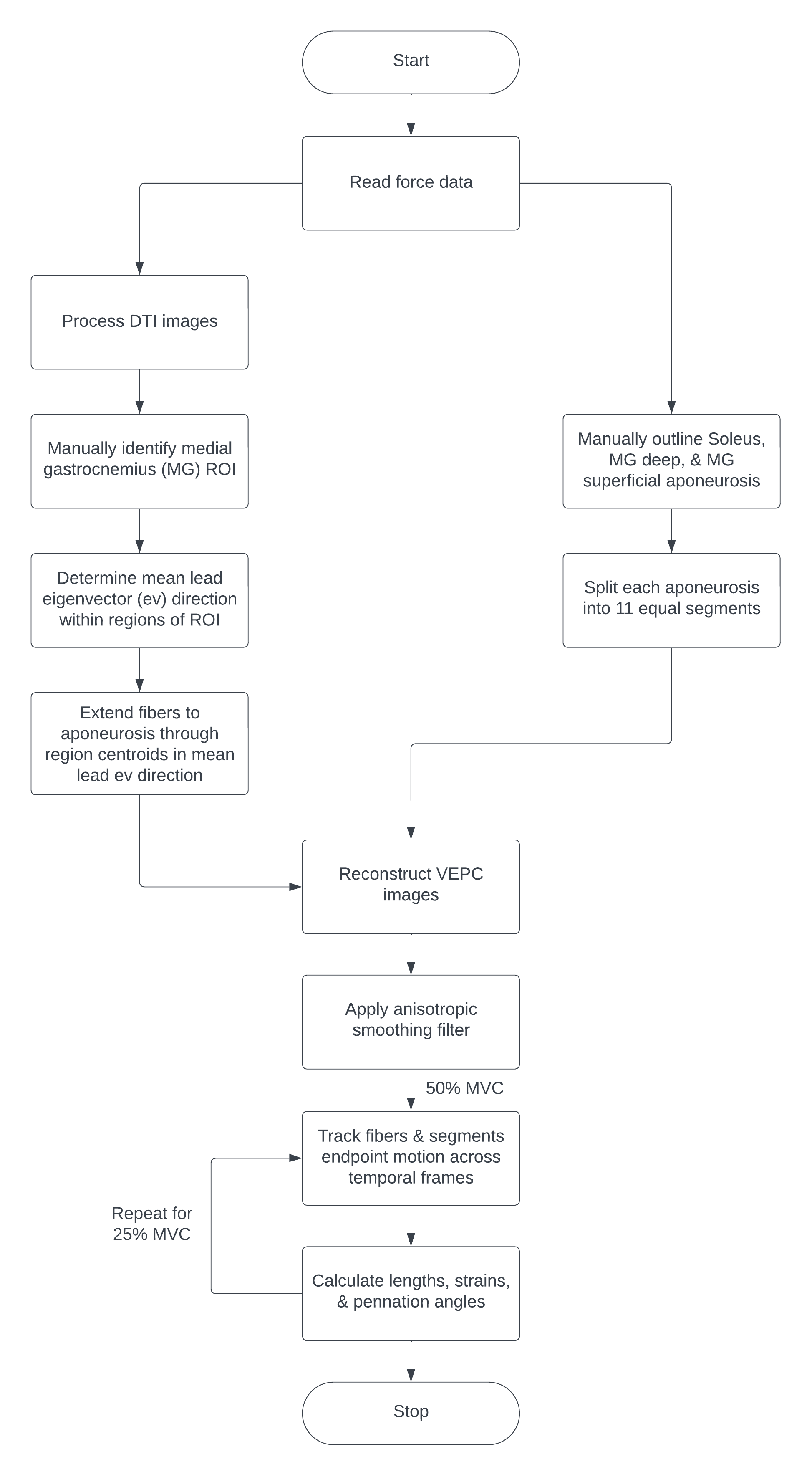


**Supplementary Figure F3:** The image processing flowchart to identify fiber directions from DTI and track the fibers and aponeurosis segments using VE-PC data to calculate fiber length, pennation angle, and strains.

**Supplemental Videos**

**Supplemental Video V1:** Video of the FSE images stepping from the beginning of the stack to the end of the stack. Included within this stack is the magnitude and phase images of the VE-PC slice; this slice is positioned at its anatomical location within the stack. The video provides an idea of the anatomical context of the VE-PC slice.

**Supplemental Video V2:** The target waveform is shown as a half-sinusoidal curve in red, the yellow line is the current %MVC for the dynamic study, the curve in blue color is a real-time plot of the force generated by the participant, the green vertical line is the trigger for the acquisition. The plot shown in the figure is projected onto a screen over the scanner face to provide real-time feedback to the subject.

**Supplemental Video V3:** Cine images of the fibers with the motion of the fiber end points tracked through the dynamic cycle for one participant for the two %MVCs and at the three ankle angles. The white solid lines are the fibers identified from the diffusion tensor images and are representative for each region (distal, middle, and proximal). Strain was calculated only for the fibers shown in this video and not for the entire region.

**Supplemental Video V4:** Cine images of the aponeurosis segments with the motion of the segment end points tracked through the dynamic cycle for one participant for the two %MVCs and at the three ankle angles. The white solid lines join the segmental end points identified along the deep and superficial aponeuroses.
