# Supplementary figures and images for: Effect of different ankle joint positions on medial gastrocnemius muscle fiber strains during isometric plantarflexion
Source: Sci Rep. 2023 Sep 11;13:14986. doi: 10.1038/s41598-023-41127-z (PMC10495375; doi:10.1038/s41598-023-41127-z)

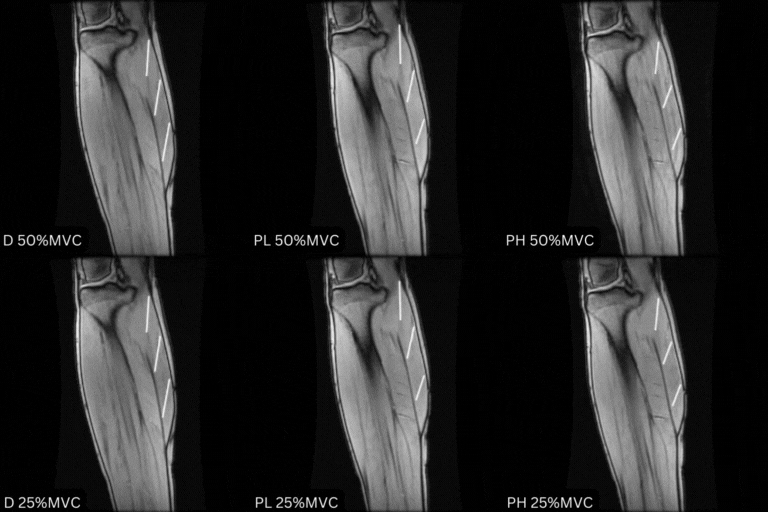

Supplement: Supplementary file 4 — Supplementary Video 3. [file 41598_2023_41127_MOESM4_ESM.gif]

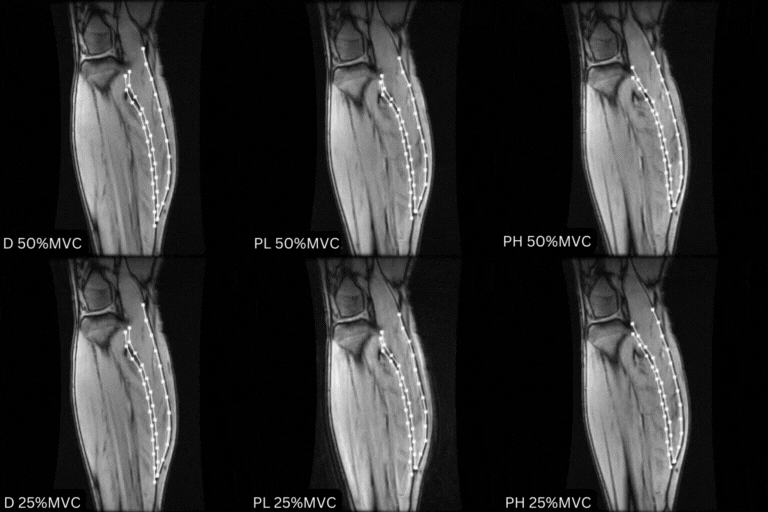

Supplement: Supplementary file 5 — Supplementary Video 4. [file 41598_2023_41127_MOESM5_ESM.gif]
